# Systemic immune-inflammation index: A new marker in differentiation of different thyroid diseases

**DOI:** 10.1097/MD.0000000000034596

**Published:** 2023-08-04

**Authors:** Selahattin Vural, Ali Muhtaroğlu, Mert Güngör

**Affiliations:** a Department of General Surgery, Faculty of Medicine, Giresun University, Giresun, Turkey; b Department of General Surgery, Faculty of Medicine, Giresun University, Giresun, Turkey.

**Keywords:** cancer, goiter, marker, systemic immune-inflammation index, thyroid

## Abstract

Inflammation plays an important role in the pathogenesis of many cancer types and is associated with thyroid malignancy. The systemic immune-inflammation index (SII) is a new inflammation marker that can be calculated from routine complete blood count (CBC). This study investigated the association between SII, a marker derived from routine CBC, and different thyroid diseases. The objective was to determine if this simple inflammation marker can distinguish between benign and malignant thyroid diseases. The medical records of all patients who underwent surgical treatment for thyroid disease between January 2018 and January 2022 were systematically evaluated. The routine preoperative CBC parameters’ demographic, clinical, and laboratory data were recorded. A total of 241 patients were included in the study, and the patients were grouped as having multinodular goiter (n = 125), lymphocytic thyroiditis (n = 44), and papillary thyroid carcinoma (PTC) (n = 73) according to pathological results. The SII was defined as the ratio of the total count of neutrophils × platelets divided by the lymphocyte count. Subgroup analysis of patients was performed according to the presence of follicular variant or thyroiditis, micro or macro carcinoma, or bilaterality of the tumor. The SII level was significantly higher in the PTC group than in the lymphocytic thyroiditis and multinodular goiter groups (*P* < .001). When we grouped the patients according to the presence of PTC as benign or malignant, the optimum cutoff point for SII level was found 654.13, with 73.8% sensitivity and 72.3% specificity from ROC analysis. In the subgroup analysis of patients with PTC, the SII level was similar according to the clinicopathological characteristics of the tumor. The differential diagnosis of thyroid diseases is important for patient management. We found that preoperative SII levels were significantly elevated in patients with PTC compared to those with benign thyroid disorders, and this simple marker can be used for the differentiation of benign and malignant thyroid disease.

## 1. Introduction

Thyroid diseases are common in the general population, and thyroid nodules with multinodular goiter (MNG) (enlargement of the thyroid gland with multiple nodules) and lymphocytic thyroiditis (LT) caused by an autoimmune process are thought to be benign disorders of the thyroid gland with a low risk of malignancy.^[1]^ Although MNG is thought to be a benign disorder, the diagnostic capacity of fine-needle aspiration may be lower due to the presence of multiple nodules, and recent studies have shown that the risk of malignancy may be higher than historically thought.^[2,3]^ Thyroid cancer is the most common endocrinological cancer,^[4]^ and papillary thyroid carcinoma (PTC).^[5]^ Studies have shown that PTC is closely related to inflammation, and patients with LT have an increased incidence of PTC.^[6]^ The co-occurrence of LT and PTC has been repeatedly reported in literature.^[7]^ Histopathology is the gold standard for differentiating between thyroid disorders.^[8]^

Inflammation plays a role in cancer pathogenesis, including proliferation, angiogenesis, and metastasis, through multiple mechanisms and pathways. Recent studies have reported that many serum systemic inflammatory response markers, such as the neutrophil-to-lymphocyte ratio (NLR), platelet-to-lymphocyte ratio, and lymphocyte-to-monocyte ratio, can be used as clinical prognostic markers in many types of cancer and thyroid cancer.^[9,10]^ The systemic immune-inflammation index (SII), defined as neutrophils × platelets/lymphocytes, is a new marker that shows patients’ inflammatory and immune status and can be easily calculated from routine blood tests. In recent studies, the SII level was found to be associated with many types of cancer, and studies have shown that the SII can be used as a prognostic marker in solid tumors.^[11]^ However, little is known about its use in thyroid diseases.

We investigated whether there is a relationship between serum SII levels and different thyroid diseases and whether this marker can be used for the differentiation of different thyroid pathologies.

## 2. Materials and methods

This retrospective study was conducted at the General Surgery Department of the Giresun University Medicine Faculty. The medical records of all patients who underwent surgical treatment for thyroid disease between January 2018 and January 2022 were systematically evaluated. Demographic and clinical data, including age; sex; laboratory data of routine preoperative complete blood count parameters, including neutrophil, lymphocyte, and platelet counts; and thyroid hormone and thyrotropin levels, were obtained from patient records. Patients with chronic diseases, such as endocrinological, urogenital, cardiovascular, gastrointestinal, immunological, or oncological diseases, and those with infectious diseases or recurrent thyroid surgery were excluded from the study. The histopathological results were obtained from the pathology department. Patients with malignant pathologies other than PTC were excluded from the study. In the subgroup analyses, patients with PTC were divided into three groups: PTC with thyroiditis, PTC with follicular variant, and normal PTC type. PTC with maximum tumor diameter ≤ 10 mm was considered as papillary thyroid microcarcinoma and > 10 mm was considered as papillary thyroid macrocarcinoma. Tumor bilaterality was also recorded from the pathology results, and 241 patients were included in the study. Patients were classified into three groups according to their pathology results: MNG (n = 125), LT (n = 44), and PTC (n = 73). The SII was defined as the ratio of the total count of neutrophils × platelets divided by the lymphocyte count. This clinical study was approved by the ethics committee of Ordu University Hospital and was performed in accordance with the Declaration of Helsinki.

### 2.1. Statistical analysis

The Statistical Package for the Social Sciences (SPSS) program (version 24.0; SPSS Inc., Chicago, IL) was used for the analysis. Descriptive statistics were used, and continuous variables were presented as means ± standard deviation, while categorical variables were presented as frequencies or percentages. The Kolmogorov–Smirnov test was used for distribution and variance homogeneity. Parameters with a normal distribution were com-pared between 2 groups by independent sample *t* test; parameters with non-normal distribution between two groups were compared with “Mann–Whitney *U*” test. Parameters with normal distribution were compared between three or more independent groups by ANOVA, and parameters with non-normal distribution between groups were compared using the Kruskal–Wallis *H* test. Bonferroni correction was applied for pairwise comparison of variables with significant differences between three or more groups. receiver operating characteristic (ROC) curve was constructed for SII area under the ROC curve value with 95% confidence interval calculated. The optimal cutoff value for the SII was determined, and sensitivity and specificity were calculated to predict benign and malignant thyroid disease. Statistical significance was less than .05.

## 3. Results

There was no statistically significant difference in the mean age between the groups. female ratio was higher in the LT group (97.7%) than in the MNG (76.8%) and PTC (76.7%) groups (*P* = .006). There was also no significant difference in the WBC and platelet counts between the groups. Neutrophil counts were significantly higher in the PTC patients (6.54 ± 2.50) than in the MNG (4.59 ± 1.86) and LT (4.41 ± 1.69) patients (4.59 ± 1.86, 4.41 ± 1.69, respectively) (*P* < .001). Lymphocyte counts were significantly lower in the PTC group (2.03 ± 0.61) than in the LT and MNG groups (2.39 ± 0.58, 2.33 ± 0.69, respectively) (*P* < .001). The demographic characteristics and laboratory data are shown in Table 1.

**Table 1:** Demographic and laboratory characteristics of study groups.

Variables	MNG (n = 125)	LT (n = 44)	PTC (n = 73)	*P* value
Age (yr)	56.78 ± 12.92	56.23 ± 10.97	56.12 ± 11.07	.922†
Gender (%)				
Female	96 (76.8)	43 (97.7)	56 (76.7)	.006*
Male	29 (23.2)	1 (2.3)	17 (23.3)	
WBC (10^3^/μL)	7.71 ± 2.56	7.49 ± 1.97	7.26 ± 1.95	.537‡
Platelet (10^3^/μL)	261.18 ± 69.43	260.65 ± 64.24	259.74 ± 53.72	.989†
Neutrophil (10^3^/μL)	4.59 ± 1.86	4.41 ± 1.69	6.54 ± 2.50	<.001*‡
Lymphocyte (10^3^/μL)	2.33 ± 0.69	2.39 ± 0.58	2.03 ± 0.61	<.001*‡
Glucose (mg/dL)	107.29 ± 33.79	110.66 ± 36.76	112.18 ± 34.61	.481‡
Urea	28.35 ± 10.14	26.47 ± 9.89	28.66 ± 8.27	.269‡
Creatinine (mg/dL)	0.70 ± 0.18	0.78 ± 0.76	0.71 ± 0.17	.373‡
ALT (IU/L])	18.39 ± 9.91	18.4 ± 7.33	19.88 ± 10.05	.400‡
AST (IU/L])	19.89 ± 6.19	20.45 ± 7.12	19.25 ± 4.51	.700‡
Na (meq/L)	141.97 ± 1.78	142.16 ± 2.11	141.82 ± 1.97	.802‡
K (meq/L)	4.54 ± 0.38	4.61 ± 0.33	4.53 ± 0.36	.466†
Cl (meq/L)	102.40 ± 8.67	103.33 ± 2.17	102.82 ± 2.45	.580‡
T4 (ng/dL)	1.23 ± 0.27	1.23 ± 0.24	1.26 ± 0.28	.582‡
TSH (mIU/L)	1.41 ± 1.61	1.71 ± 1.53	1.70 ± 1.45	.209‡
SII	543.30 ± 305.21	512.69 ± 251.09	920.28 ± 496.35	<.001*‡

Data expressed as mean ± standard deviation.

ALT = alanine aminotransferase, AST = aspartate aminotransferase, LT = lymphocytic thyroiditis, MNG = multinodular goiter, PTC = papillary thyroid carcinoma, SII = systemic inflammation index, TSH = thyroid-stimulating hormone, WBC = white blood cell.

* Statistical significance.

† ANOVA test.

‡ Kruskal–Wallis *H* test.

**Figure 1.**
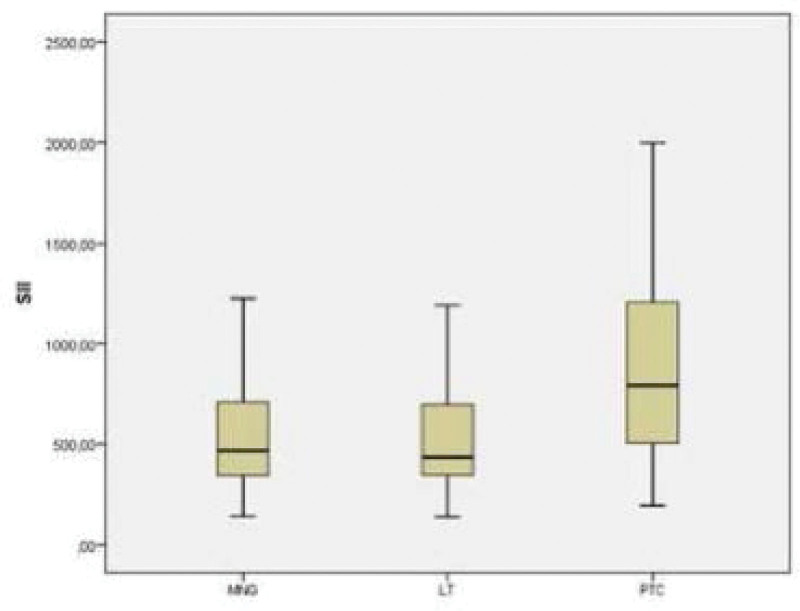
Comparison of mean SII levels between groups. SII = systemic immune-inflammation index.

Mean SII levels were significantly higher in the PTC group (920.28 ± 496.35) than in the LT (512.69 ± 251.09) and MNG groups (543.30 ± 305.21) (*P* < .001). A comparison of SII levels in all groups is shown in Figure 1.

**Figure 2.**
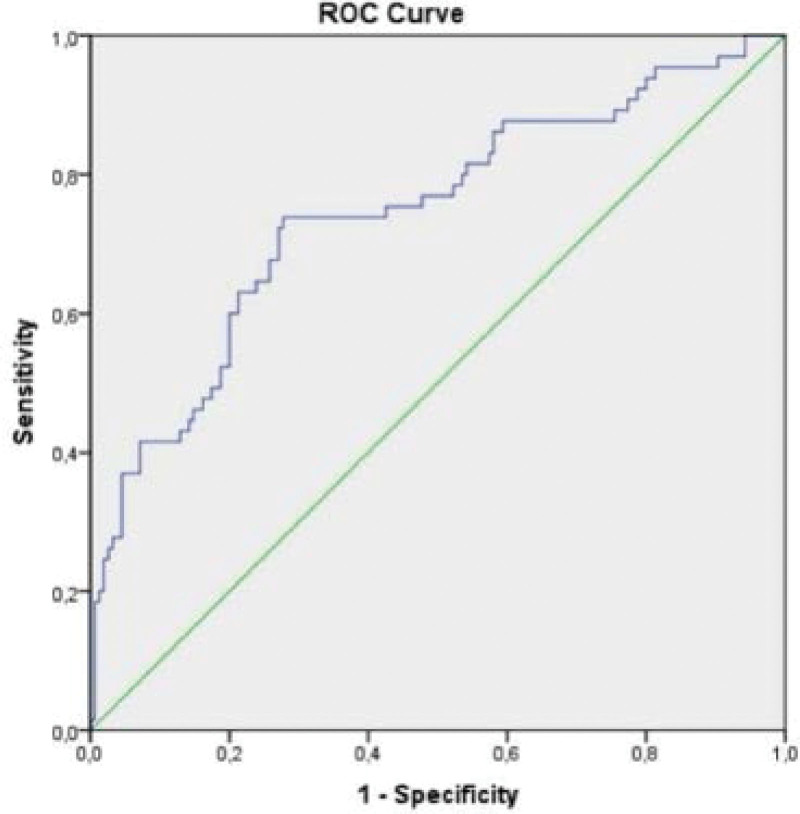
ROC curve for SII at cutoff value of 654.13. ROC = receiver operating characteristic, SII = systemic immune-inflammation index.

Histopathological diagnosis was used for the presence of malignancy, and according to the presence of malignancy, the MNG and LT groups were defined as benign, and the PTC group was defined as malignant. ROC curve analysis suggested that the optimum cutoff point for SII was 654.13 with 73.8% sensitivity and with 72.3% specificity for discrimination of benign and malignant thyroid disease. The area under the ROC curve value for SII was 0.74 (95% confidence interval: 0.69–0.82). The ROC curve for the SII for the discrimination of benign and malignant thyroid disease is shown in Figure 2.

In subgroup analysis, in the PTC group, the mean SII level was not significantly different between micropapillary and macropapillary carcinomas and was also similar according to the bilaterality of the tumors. Finally, preoperative SII levels were not found to be associated with PTC subtypes, such as the presence of thyroiditis and follicular variants. The association of SII and NLR with the clinicopathological characteristics of PTC is shown in Table 2.

**Table 2:** SII level according to clinicopathological characteristics of PTC.

Variables	SII level, mean ± SD	*P* value
Subtype		
Thyroiditis (n = 14)	875.02 ± 494.11	.311*
Follicular variant(n = 5)	1221.75 ± 504.16	
Normal (n = 54)	900.73 ± 496.10	
Tumor size		
Micropapiller type (n = 46)	947.95 ± 512.67	.593†
Macropapiller type (27)	869.75 ± 471.94	
Bilaterality		
Yes (n = 37)	924.69 ± 521.08	.823†
No (36)	915.45 ± 476.28	

PTC = papillary thyroid carcinoma, SII = systemic inflammation index.

* Kruskal–Wallis *H* test.

† Mann–Whitney *U* test.

## 4. Discussion

Chronic inflammation is considered to play a role in the pathogenesis of several diseases including cancer, cardiovascular disease, diabetes, and metabolic syndrome.^[12]^ In the pathogenesis of cancer, inflammation has been shown to have a role both in cancer development and progression. This relationship has been clearly reported, especially for gastrointestinal malignancies such as gastric cancer due to H. pylori, hepatocellular carcinoma due to viral hepatitis, and colorectal cancer due to idiopathic inflammatory bowel disease.^[13]^ The relationship between inflammation and cancer is not clearly defined, but inflammatory cells and mediators may cause changes in cell function, and inflammation may have a carcinogenic or anti-carcinogenic effect through tumor and host interactions.^[14]^

The most common endocrine malignancy is thyroid cancer, and the major type is the papillary type.^[15]^ In terms of thyroid diseases, the association between chronic inflammation and PTC was first demonstrated by Dailey in 1955.^[16]^ In other studies, a positive association between chronic inflammation and PTC has been found, showing that patients with thyroiditis have an increased risk of PTC, but paradoxically, the presence of thyroiditis in papillary thyroid cancer has been found to be associated with a better prognosis.^[17]^ However, the role of inflammation in the pathogenesis of thyroid cancer is not well defined in the literature.

Many studies have shown an association between simple serum hematological markers, such as total and differential leukocyte counts, and different cancer types. Moreover, studies have shown that these markers can be used as prognostic markers for these diseases.^[18]^ Although serum inflammatory markers have been found to be independent prognostic factors in many cancer types, they are not considered in prognostic or staging systems.^[19]^ Recently, the SII was developed as a new index marker based on both circulating immune and inflammatory cells, such as platelets, neutrophils, and lymphocytes, and is considered to simultaneously show both the inflammatory and immune status of patients.^[11]^

Increased neutrophil or platelet counts and/or decreased lymphocyte counts increase SII levels. Neutrophils are important mediators of immunity in acute inflammation, and neutrophilia has long been known as a marker of systemic inflammatory response.^[19]^ Recent studies have also shown that neutrophils play a role in carcinogenesis. Increased neutrophils in cancer may be due to the ectopic production of growth factors as part of a paraneoplastic syndrome or inflammation due to tissue destruction and cytokine release.^[20,21]^ In vitro and in vivo studies have shown that neutrophils stimulate tumor growth, progression, and metastasis, especially because of secreted cytokines and growth factors.^[18]^ Teramukai et al^[22]^ reported that increased levels of circulating neutrophils are associated with an adverse prognosis in patients with cancer. In terms of thyroid cancer, Galdiero et al^[23]^ showed that neutrophils are activated in thyroid cancer with upregulation of CD11b and CD66b and that levels were correlated with tumor size.

In our study, we found that neutrophil levels were significantly higher in patients with PTC than in patients with LT and MNG. In contrast, lymphocytes cause cytotoxic cell death and inhibit tumor cell proliferation; therefore, they are known to suppress tumor growth and progression in patients with cancer.^[24]^ Low lymphocyte counts have been associated with generalized suppression of the immune system in patients with cancer.^[25]^ Vasant et al^[26]^ reported that low lymphocyte levels may activate reactive oxidatives species-mediated apoptosis; therefore, lymphocyte infiltration may have a protective role in thyroid cancer development. In our study, lymphocyte counts were lower in patients with PTC than those with benign PTC. Platelets are thought to form a shield around tumor cells, increase their capacity for adhesion, and prevent cell death. They can also secrete transforming growth factor–beta, vascular endothelial growth factor, and platelet derived growth factor, which lead to tumor progression and metastasis.^[27]^

There were no significant differences in the platelet counts between the groups in our study. Thus, a high SII may represent high levels of neutrophils and platelets with low levels of lymphocytes, usually indicating a stronger inflammatory response and weaker immune response in cancer patients.^[11]^ Hu et al^[28]^ first reported the association between SII and hepa-tocellular carcinoma and found that the index was also associated with disease prognosis, vascular invasion, tumor size, and recurrence. Other studies have shown that SII can be used to predict the prognosis of other solid tumors.^[11,29]^ Geng et al^[30]^ reported in their study that SII was a more objective marker of host inflammatory and immune response status compared to other systemic inflammation index markers such as platelet-to-lymphocyte ratio and NLR.

In recent studies, hematological markers have also been studied in thyroid disease and have been found to be associated with malignancy. Kocer et al^[14]^ reported that the optimum NLR level was 1.91 with 89.0% sensitivity and 54.5% specificity, and that it can be used as a potential marker for discrimination of benign and malignant thyroid disease. NLR was also found to be associated with larger tumor size, multifocality, and a higher stage of PTC.^[16]^ In a meta-analysis, Feng et al^[31]^ assessed the association between pretreatment NLR and prognosis and clinicopathological features in thyroid cancer patients and showed that increased NLR was correlated with larger tumor size, positive lymph nodes, and distant metastasis. Although there have been studies on NL and thyroid disease, little is known about SII and its association with thyroid pathologies. In our study, we found that SII level was significantly higher in patients with PTC, and that 73.8% sensitivity and 72.3% specificity with a level of 654.13 can be used to discriminate between benign and malignant diseases. The results of studies on SII and cancer progression have been inconsistent in the literature. In many recent studies, elevated SII was found to be associated with poor overall survival in many types of cancer, while other studies reported no association between overall survival and SII.^[11]^ Zhong et al^[11]^ found a significant association between an elevated SII and poor prognosis in solid tumors. In terms of thyroid disease, Zhang et al^[32]^ reported that the SII level was independently associated with central lymph node metastasis in clinically nodal-negative PTC. In our study, in subgroup analyses of PTC patients, SII levels were not found to be correlated with bilaterality, micro-or macrocarcinoma, or tumor subtype.

A limitation of our study was its retrospective design and that our study could not define the pathogenesis of higher SII level in patients with PTC. Further prospective studies with a larger study population are needed to confirm our results, and a unified cutoff value for the SII is needed to discriminate malignant thyroid disease.

## 5. Conclusions

To the best of our knowledge, this is the first study to investigate the value of the SII for the discrimination of benign and malignant thyroid diseases. The differential diagnosis of thyroid disease is important for the management of patients, and we found that preoperative SII levels were significantly elevated in patients with PTC compared to those with benign thyroid disorders, and that this simple and inexpensive marker can be used for differentiation of benign and malignant thyroid disease. However, in patients with PTC, the SII level was similar according to the clinicopathological features of the tumors.

## Author contributions

**Conceptualization:** Selahattin Vural.

**Data curation:** Selahattin Vural.

**Formal analysis:** Selahattin Vural.

**Investigation:** Selahattin Vural.

**Methodology:** Selahattin Vural.

**Project administration:** Mert Güngör.

**Software:** Mert Güngör.

**Supervision:** Selahattin Vural.

**Validation:** Mert Güngör.

**Visualization:** Mert Güngör.

**Writing – original draft:** Selahattin Vural.

**Writing – review & editing:** Selahattin Vural, Ali Muhtaroğlu.
